# Metabotropic Glutamate_2_ Receptors Play a Key Role in Modulating Head Twitches Induced by a Serotonergic Hallucinogen in Mice

**DOI:** 10.3389/fphar.2018.00208

**Published:** 2018-03-15

**Authors:** Mark J. Benvenga, Stephen F. Chaney, Melvyn Baez, Thomas C. Britton, William J. Hornback, James A. Monn, Gerard J. Marek

**Affiliations:** ^1^Neuroscience Discovery Research, Lilly Research Laboratories, Eli Lilly and Company, Indianapolis, IN, United States; ^2^Lilly Corporate Center, Eli Lilly and Company, Indianapolis, IN, United States; ^3^Discovery Chemistry Research, Lilly Research Laboratories, Eli Lilly and Company, Indianapolis, IN, United States

**Keywords:** 5-hydroxytryptamine_2A_ (5-HT_2A_) receptors, DOI, CBiPES, LY379268, LY341495, head twitches, prefrontal cortex (PFC), transgenic mice

## Abstract

There is substantial evidence that glutamate can modulate the effects of 5-hydroxytryptamine_2A_ (5-HT_2A_) receptor activation through stimulation of metabotropic glutamate_2/3_ (mGlu_2/3_) receptors in the prefrontal cortex. Here we show that constitutive deletion of the mGlu_2_ gene profoundly attenuates an effect of 5-HT_2A_ receptor activation using the mouse head twitch response (HTR). MGlu_2_ and mGlu_3_ receptor knockout (KO) as well as age-matched ICR (CD-1) wild type (WT) mice were administered (±)1-(2,5-dimethoxy-4-iodophenyl)-2-aminopropane (DOI) and observed for head twitch activity. DOI failed to produce significant head twitches in mGlu_2_ receptor KO mice at a dose 10-fold higher than the peak effective dose in WT or mGlu_3_ receptor KO mice. In addition, the mGlu_2/3_ receptor agonist LY379268, and the mGlu_2_ receptor positive allosteric modulator (PAM) CBiPES, potently blocked the HTR to DOI in WT and mGlu_3_ receptor KO mice. Conversely, the mGlu_2/3_ receptor antagonist LY341495 (10 mg/kg) increased the HTR produced by DOI (3 mg/kg) in mGlu_3_ receptor KO mice. Finally, the mGlu_2_ receptor potentiator CBiPES was able to attenuate the increase in the HTR produced by LY341495 in mGlu_3_ receptor KO mice. Taken together, all of these results are consistent with the hypothesis that that DOI-induced head twitches are modulated by mGlu_2_ receptor activation. These results also are in keeping with a critical autoreceptor function for mGlu_2_ receptors in the prefrontal cortex with differential effects of acute vs. chronic perturbation (e.g., constitutive mGlu_2_ receptor KO mice). The robust attenuation of DOI-induced head twitches in the mGlu_2_ receptor KO mice appears to reflect the critical role of glutamate in ongoing regulation of 5-HT_2A_ receptors in the prefrontal cortex. Future experiments with inducible knockouts for the mGlu_2_ receptor and/or selective mGlu_3_ receptor agonists/PAMs/antagonists could provide an important tools in understanding glutamatergic modulation of prefrontal cortical 5-HT_2A_ receptor function.

## Introduction

Metabotropic glutamate receptors (mGluRs) have been implicated in a wide variety of effects with clinical implications. The group II mGlu_2_ and mGlu_3_ receptor subtypes have been shown in a wide range of animal studies to have effects in models predictive of antidepressant activity (Chaki et al., 2004; Yasuhara et al., 2006; Nikiforuk et al., 2010; Fell et al., 2011), anxiolytic activity (Klodzinska et al., 2002; Schoepp et al., 2003; Galici et al., 2006) and antipsychotic activity (Moghaddam and Adams, 1998; Johnson et al., 2005; Rorick-Kehn et al., 2007). These preclinical predictions have been confirmed for orthosteric mGlu_2/3_ receptor agonists in the treatment of schizophrenia, though in a limited subpopulation of patients (Patil et al., 2007; Kinon et al., 2015). In contrast to schizophrenia, safety considerations from preclinical data available at that time precluded registration of the first highly potent orally available orthosteric mGlu_2/3_ receptor agonist demonstrated to be effective in patients with generalized anxiety disorder (Schoepp et al., 2003; Michelson et al., 2005; Dunayevich et al., 2008).

Suppression of rodent head twitches induced by serotonergic hallucinogens via 5-hydroxytryptamine_2A_ (5-HT_2A_) receptor activation appears to be a promiscuous preclinical pharmacological screen sensitive to both antipsychotic drugs and many antidepressant drugs (Canal and Morgan, 2012; Marek, 2017). Previous studies have shown that orthosteric mGlu_2/3_ receptor agonists suppress 2,5-dimethoxy-4-iodoamphetamine (DOI)-induced head twitches in both rats and mice (Gewirtz and Marek, 2000; Klodzinska et al., 2002; Gonzalez-Maeso et al., 2008; Moreno et al., 2011, 2012; Wieronska et al., 2013). A permissive role of the mGlu_2_ receptor in these effects is supported by several studies where mGlu_2_ receptor positive allosteric modulators (PAMs) also suppressed head twitches induced by phenethylamine hallucinogens (Benneyworth et al., 2007; Lavreysen et al., 2015; Griebel et al., 2016).

Head twitches induced by 5-HT_2A_ receptor activation are supported by direct infusions of serotonergic hallucinogens into the medial prefrontal cortex (mPFC) of the rat (Willins and Meltzer, 1997). DOI-induced head twitches, in addition to suppression by mGlu_2/3_ receptor agonists or mGlu_2_ receptor PAMs, also are suppressed by activation of mGlu_4_, adenosine A_1_, and m-opioid receptors (Marek, 2003, 2009; Wieronska et al., 2013). Interestingly, these G_i_/G_o_-coupled receptor agonists/PAMs also suppress excitatory postsynaptic currents/potentials (EPSCs/EPSPs) induced by activation of 5-HT_2A_ receptors in layers I and Va of the mPFC/neocortex (Marek and Aghajanian, 1998; Stutzman et al., 2001; Zhang and Marek, 2007; Slawinska et al., 2013). Thus, 5-HT and serotonergic hallucinogen acts on mPFC layer V pyramidal cells by inducing glutamate release with DOI-induced head twitches as one salient behavioral manifestation (Marek, 2017).

Lesion studies have also suggested that the intralaminar and midline thalamic nuclei, the non-specific thalamic nuclei known to be important in arousal and attention, may be the source of neurons from which 5-HT_2A_ receptor stimulation induces glutamate release onto cortical layer V pyramidal cells (Lambe and Aghajanian, 2001; Marek et al., 2001). These findings are in keeping with (1) the higher expression of mGlu_2_ mRNA in the thalamic midline and intralaminar nuclei compared to other thalamic nuclei or the prefrontal cortex/neocortex (Ohishi et al., 1993, 1998); (2) the predominant physiological role of mGlu_2_ receptors as autoreceptors (Marek et al., 2000; Schoepp, 2001); (3) the laminar overlap within the prefrontal cortex of relatively higher densities of 5-HT_2A_ and mGlu_2_ receptor binding in layers I and Va (Marek et al., 2000, 2001; Schoepp, 2001; Wright et al., 2013); and (4) the known termination of thalamic midline and intralaminar thalamic nuclei in layers I and Va (Berendse and Groenewegen, 1991; Van der Werf et al., 2002). There undoubtedly are effects of 5-HT_2A_ receptor activation independent of this thalamocortical pathway (Celada et al., 2008; Wischhof and Koch, 2016). However, increased thalamocortical connectivity appears to play a role in psychotomimetic effects of LSD and psilocybin in humans (Carhart-Harris et al., 2013; Tagliazucchi et al., 2016; Muller et al., 2017). But given the role of these thalamic nuclei in attention and arousal, coupled with massive feedback from layer V pyramidal neurons as drivers of intralaminar and midline thalamic nuclei activity (Saalmann, 2014), this thalamocortical circuitry likely plays a major role on psychotomimetic effects of serotonergic hallucinogens.

In the context of the pharmacology, circuitry and potential clinical implications of modulating the DOI-induced head twitch response (HTR), we applied genetic strategies studying both mGlu_2_ and mGlu_3_ knockouts to understand the role of mGlu_2_ and mGlu_3_ receptors in mediating the effects of mGlu_2/3_ receptor agonists or mGlu_2/3_ receptor antagonists in modulating DOI-induced head twitches. One question of interest was the contributions of mGlu_2/3_ receptors to the DOI-induced head twitch model. A second question was whether modulation of mGlu_2_ and/or mGlu_3_ receptors would be therapeutic drug candidates. The primary caveat to this approach is that the mGlu_2_ receptor appears to play an autoreceptor role in modulating DOI-induced head twitches. As discussed earlier, acute or single dose administration of mGlu_2/3_ receptor agonists suppress while mGlu_2/3_ receptor antagonists enhance DOI-induced head twitches, consistent with the widespread role of mGlu_2_ receptors as an autoreceptor on glutamatergic terminals/pre-terminal axons. Thus, this key homeostatic role played by an autoreceptor raises the possibility that differential regulation of DOI-induced head shakes could be observed with acute (single dose) vs. chronic (daily administration). A mGlu_2_ constitutive knockout would in and of itself constitute a chronic perturbation of the system existing throughout the life of the mutant mouse.

Even at the time these studies were begun, evidence for potential glutamatergic modulation of prefrontal cortical 5-HT_2A_ receptors was present. First, cortical 5-HT_2A_ receptor binding was increased by chemical thalamic lesions (Marek et al., 2001). Second, two daily mGlu_2/3_ agonist systemic doses prior to treatment with DOI prevented down-regulation of DOI receptor binding in the prefrontal cortex (Marek et al., 2006). Subsequent to these studies, chronic administration of a mGlu_2/3_ receptor antagonist LY341495 on a daily basis for 21 days decreased both 5-HT_2A_ receptor binding and LSD-induced head twitches in mice (Moreno et al., 2013). This latter result is completely opposite of a previous finding that a single dose of the same mGlu_2/3_ receptor antagonist increased DOI-induced head twitches in rats (Gewirtz and Marek, 2000). Not surprisingly in the light of these caveats, paradoxical effects with the mGlu_2_ receptor knockout mice were observed in this manuscript where a dramatic rightward shift in the DOI-induced head twitch dose response. This was in contrast to the lack of any apparent change in the DOI dose-response relationship with the mGlu_3_ knockout mice. These transgenic mouse studies of the DOI dose-response relationship support a prominent role for mGlu_2_ receptors in modulating DOI-induced head twitches. The lack of an attenuation of the acute effects of either an mGlu_2/3_ receptor agonist, an mGlu_2_ receptor PAMs, or mGlu_2/3_ receptor antagonists in mGlu_3_ receptor KO mice in this manuscript is consistent with a potentially necessary and sufficient role for the mGlu_2_ receptor in modulating DOI-induced head twitches for group II mGlu receptor ligands. Thus, mGlu_2_ receptors appear to play a major modulatory role in the circuitry controlling DOI-induced head twitches while screening with this behavioral model also suggests mGlu_2_ receptor agonists and PAMs as candidate antipsychotic treatments.

## Materials and Methods

### Animals

MGlu_2_ and mGlu_3_ receptor knockout mouse strains were generated by homologous recombination as previously described (Linden et al., 2005). Wild type (WT) ICR (CD-1) littermates were used in all experiments employing KO animals. In all experiments using KO mice, animals were used in more than one experiment, with at least a 7-day washout period. A total of 32 mGlu_2_ receptor KO mice and 40 mGlu_3_ receptor KO mice were used in these experiments (Taconic Inc., Germantown, NY, United States). Other experiments utilized ICR (CD-1) out bred mice (*n* = 104; Harlan Inc., Indianapolis, IN, United States). The mice used in these experiments were 4–8 weeks old. Mice were housed 6–10 per cage under a 12:12 light-dark cycle (lights on at 6 a.m.), and food and water were available at all times. All animal protocols were approved by the Eli Lilly and Company Animal Care and Use Committee and followed guidelines recommended by NIH at the time the experiments were conducted.

### Head Twitch Test

The test apparatus had eight individual observation chambers and was made of Plexiglas, measuring 10 cm × 10 cm × 12 cm per chamber, through which all animals were observed. An experienced blinded observer watched eight animals in tandem and recorded all head twitches on a multiple counter (Fisher Scientific, Inc., Pittsburgh, PA, United States). Mice were brought from the colony room in groups of eight, weighed, and allowed to acclimate to the test chamber for 15 min prior to testing. Head twitches were induced by DOI (3 mg/kg, i.p.), and mice were observed for 30 min beginning 5 min after dosing. The only exception to this was the initial DOI dose-response determination where the WT mice observation duration was 15 min instead of 30 min. LY379268, LY341495 and CBiPES were administered i.p., 30 min prior to DOI. When CBiPES was administered with LY341495, CBiPES was dosed 5 min prior to LY341495. Each experimental group consisted of eight animals per group except for the experiment testing DOI in mGlu_3_ receptor KO animals (*N* = 10), the experiment testing the LY379268 dose-response relationship in WT mice (*N* = 6) and when LY341495 was tested in mGlu_3_ receptor KO animals (*N* = 16). The sample size used for these experiments were determined in part by availability transgenic mice. Separate groups of animals were used for each dose of DOI in all experiments with CD-1 (ICR) mice, and in all experiments utilizing KO/WT animals, mice were used at least twice, with a 7-day washout period between tests.

The methodology used for the DOI-induced HTR was fit for purpose as a relatively modest-high throughput behavioral assay for a range of drug discovery programs at the Lilly Research Laboratories. Statisticians and biologists worked in tandem to validate this *in vivo* assay (and other *in vivo* and *in vitro* assays). This validation included determining the number of animals required for individual behavioral experiments as well as the statistical tests used to evaluate whether a concentration- or dose-related effect was present. A critical part of the validation for the DOI-induced HTR including running these validating experiments with a trained observed (SFC), with substantial personal experience of measuring DOI-induced head twitches under these condition. On occasion, additional validating experiments were carried out such as an experiment where CBiPES statistically suppressed DOI-induced head twitches in a dose-related manner where a significant decrease in rat head twitches induced by DOI (3 mg/kg, i.p.) occurred for a 30 mg/kg CBiPES dose (approximately 10 head twitches with DOI alone down to about 4 head twitches with DOI/CBiPES). Furthermore, this mouse DOI-induced HTR assay was used for optimization of the physicochemical characteristics of mGlu_2_ receptor PAM SAR that later resulted in mGlu_2_ receptor PAMs that were both more potent and centrally penetrant. In a set of 12 compounds not described in this manuscript, the mean frequency of head twitches/30 min observation periods induced by DOI alone were 7.9 ± 1.2 (SD) in WT CD1 mice. The range of DOI-induced head twitches was (6.4–10.7). Only one experiment was the mean DOI-induce head twitch frequency lower than the 95% confidence intervals for the set of 12 experiments. Only for two experiments were the mean frequency of DOI-induced head twitches higher than the 95% confidence interval for the entire set of 12 experiments. MGlu_2_ receptor PAMs significantly reduced the frequency of DOI-induced head twitches in doses ranging from 1 to 30 mg/kg. Additional internal converging validity of the DOI-induced head twitch assay for the mGlu_2_ receptor PAM program was found in similar results for the same mGlu_2_ receptor PAMs with respect to potency and maximal efficacy with hyperactivity induced by a NMDA receptor antagonist. Another internal converging validity of the DOI-induced head twitch assay for the mGlu_2_ receptor PAM was found in similar results with respect to potency and maximal efficacy when testing for antidepressant-like activity in mice or rats on an operant differential-reinforcement-of-low rate (DRL) schedule of reinforcement. A range of other discovery projects (including selective mGlu_2_ receptor orthosteric agonists, mGlu_5_ receptor negative allosteric modulators, orexin receptor antagonists) also successfully utilized the DOI-induced head twitch assay either for supporting the development of new projects and/or utility as an early relatively modest-high behavioral throughput assay that was complimented by *in vivo* receptor occupancy (where available) and other physiological/behavioral assays.

### Drugs

(±)-DOI hydrochloride was obtained from a commercial source (Sigma-Aldrich, Inc., St. Louis, MO, United States) and dissolved in dH_2_O. LY379268 (1*R*,4*R*,5*S*,6*R*-4-amino-oxabicyclo[3.1.0]hexane-4,6-dicarboxylic acid), LY341495 (2*S*1′*S*,2′*S*)-2-(9-xanthylmethyl)-2-(2′carboxycyclopropyl)glycine and CBiPES (*N*-(4′-cyano-biphenyl-3-yl)-*N*-(3-pyridinymethyl)-ethanesulfonamide hydrochloride) were synthesized at Lilly Research Labs (Indianapolis, IN, United States). LY379268 and LY341495 were dissolved in dH_2_O with the addition of drops of 5 N NaOH to adjust pH to 6–7 while CBiPES was suspended in 1% methylcellulose, 0.25% Tween 80, 0.05% Dow Antifoam, dH_2_0. All compounds were administered by the intraperitoneal (i.p.) route and given in a volume of 10 ml/kg. All compounds were weighed and described in figures as the nominal weight including the associated salt.

### Statistics

The data obtained in the experiments are presented as means ± SEM and was analyzed using a one-way ANOVA, followed *post hoc* by a Dunnett’s test to determine individual difference using *p* < 0.05 for the significance level. Where a single dose of drug was compared to a vehicle condition, the Student’s *t*-test was employed using *p* < 0.05 for the significance level. In addition, all groups in each experiment were analyzed for total number of animals exhibiting head twitch behavior using the Fisher Exact Test with *p* < 0.05 for significance. All experiments were analyzed using JMP 5.1 (SAS Institute, Inc., Cary, NC, United States).

## Results

### DOI/mGlu Receptor Effects in CD-1 Mice

**FIGURE 1:**
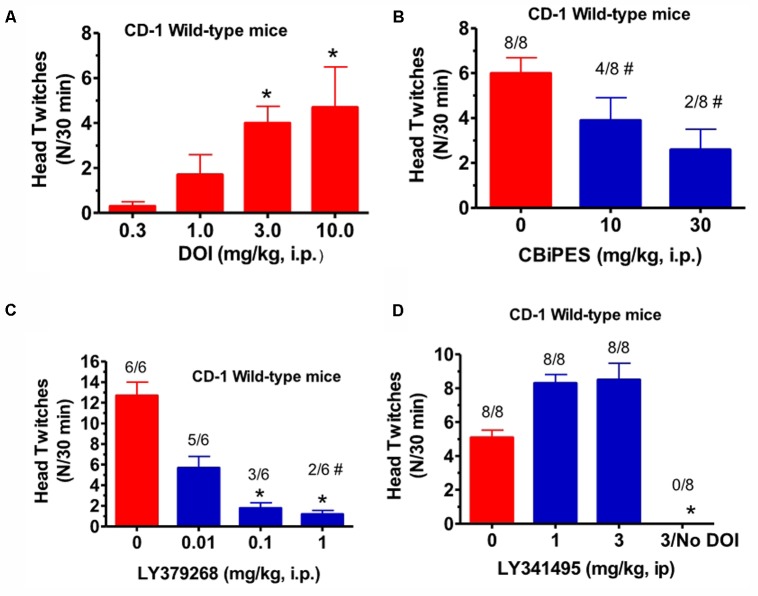
**(A)** The effect of (±)-DOI (0.3–10 mg/kg) on head twitches in CD-1 WT mice observed for 15 min following drug administration. Each bar represents the mean (±SEM) of six animals after i.p. dosing. Significantly different from 0, ^∗^*p* < 0.05. **(B)** The effect of CBiPES (10–30 mg/kg) on head twitches induced by DOI (3 mg/kg) in CD-1 WT mice observed for 30 min following drug administration. CBiPES was administered 30 min prior to DOI. Each bar represents the mean (±SEM) of eight animals after i.p. dosing. Significantly different from 0 or the vehicle condition by Fisher Exact Test, ^#^*p* < 0.05. **(C)** The effect of LY379268 (0.01–1 mg/kg) on head twitches induced by DOI (3 mg/kg) in CD-1 WT mice observed for 30 min following drug administration. LY379268 was administered 30 min prior to DOI. Each bar represents the mean (±SEM) of six animals after i.p. dosing. Significantly different from 0 (vehicle) by the Fisher Exact Test, ^#^*p* < 0.05. **(D)** The effect of LY341495 (1–3 mg/kg) on head twitches induced by DOI (3 mg/kg) in CD-1 WT mice observed for 30 min following drug administration. LY341495 was administered 30 min prior to DOI. Each bar represents the mean (±SEM) of eight animals after i.p. dosing. Significantly different from 0 (vehicle condition), ^∗^*p* < 0.05. Note that the DOI dose-response relationship is the only experiment in this paper where a 15 min observation period was employed, unlike all other experiments using a 30 min observation period.

(±)-DOI (0.3–10 mg/kg, i.p.), given alone to CD-1 mice, produced an increase in mean head twitches [*F*(3,20) = 4.09, *p* < 0.05] to 4.1 in a 15 min observation period, at a dose of 3 mg/kg (**Figure 1A**). Lower doses produced non-significant increases in head twitches, while a higher dose of 10 mg/kg produced increased variability yet a significant increase in mean head twitches. This 10 mg/kg DOI dose also produced an increase in arousal, manifested by increased locomotion in the observation cage. For this reason, 3 mg/kg (±)-DOI was chosen as the optimal dose for further testing with a 30 min observation period. The mGlu_2_ receptor PAM, CBiPES (10–30 mg/kg), when given to DOI-treated CD-1 mice, produced a mean of 2.3 head twitches at 30 mg/kg, a 60% reduction from DOI and vehicle-treated animals (**Figure 1B**). Although this dose was not statistically different from vehicle [*F*(2,21) = 0.94, *p* = 0.31], the number of animals emitting head twitches (2/8) was significantly different from vehicle/DOI-treated animals (8/8; Fisher Exact, *p* = 0.003). Additionally, the 10 mg/kg CBiPES dose also produced a significant decrease in the number of animals emitting head twitches (4/8; Fisher Exact, *p* = 0.038). LY379268 (**Figure 1C**), the orthosteric mGlu_2/3_ receptor agonist, was very potent at decreasing the mean head twitches produced by DOI [*F*(3,20) = 8.51, *p* < 0.001]. At a dose of 0.1 mg/kg, there was a significant decrease in mean head twitches to less than 15% of DOI alone. Additionally, all LY379268 doses tested (0.01–1.0 mg/kg) reduced head twitches to less than 50% of the mean number when receiving DOI alone. The mGlu_2/3_ receptor antagonist, LY341495 (1–3 mg/kg), when tested in the presence of DOI in CD-1 mice, produced a numerical increase in the mean number of head twitches as compared to DOI alone, but the effect did not reach significance [*F*(2,21) = 1.88, *p* = 0.17, **Figure 1D**].

### DOI Effects in mGlu_2_ or mGlu_3_ Receptor KO Mice

**FIGURE 2:**
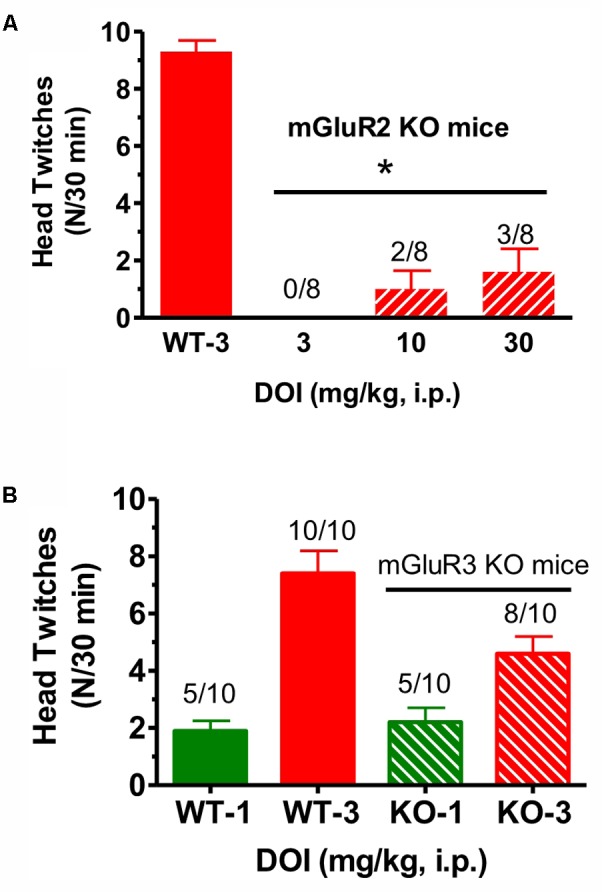
**(A)** The effect of DOI (3 mg/kg) on head twitches in WT and DOI (3–30 mg/kg) in mGlu_2_ receptor KO mice for 30 min following drug administration. Each bar represents the mean (±SEM) of eight animals after i.p. dosing. Significantly different from WT performance, ^∗^*p* < 0.05. WT, wild type; KO, knockout. **(B)** The effect of DOI (1–3 mg/kg) on head twitches in WT and the effect of DOI (1–3 mg/kg) on head twitches in mGlu_3_ receptor KO mice for 30 min following drug administration. Each bar represents the mean (±SEM) of 10 animals after i.p. dosing. WT, wild type; KO, knockout. The horizontal line is shown above the data for the mGlu_2_ receptor KO mice and for the mGlu_3_ receptor KO mice in the top **(A)** and lower figures**(B)**, respectively.

When DOI (3–30 mg/kg) was administered to mGlu_2_ receptor KO mice in the same fashion, and compared to the effect of 3 mg/kg given to littermate WT mice, there was a significant reduction in mean head twitches [*F*(3,28) = 40.5, *p* < 0.0001] (**Figure 2A**). In fact, at the highest DOI dose tested (30 mg/kg), only 3 of 8 mGlu_2_ receptor KO mice exhibited head twitches. DOI did not induce more than five head twitches at either dose level in any of the few mGlu_2_ receptor KO mice with observed head twitches. Thus, the number of DOI-induced head twitches in these mice was even below the range of the mean DOI-induced head twitches for the entire series of 12 groups of WT mice tested with 3 mg/kg described in the Section “Materials and Methods.” In contrast, when tested in mGlu_3_ receptor KO mice, DOI (1–3 mg/kg) produced mean head twitches similar to that seen in WT mice. At doses of 1 and 3 mg/kg, there was no significant difference between WT and mGlu_3_ receptor KO mice when tested with DOI (**Figure 2B**). Given the surprisingly low number of DOI-induced head twitches in the mGlu_2_ receptor KO mice, the effects of LY379268, CBiPES and LY379268 were not studied in the mGlu_2_ receptor KO mice.

### mGlu Receptor Drugs in mGlu_3_ Receptor KO Mice

**FIGURE 3:**
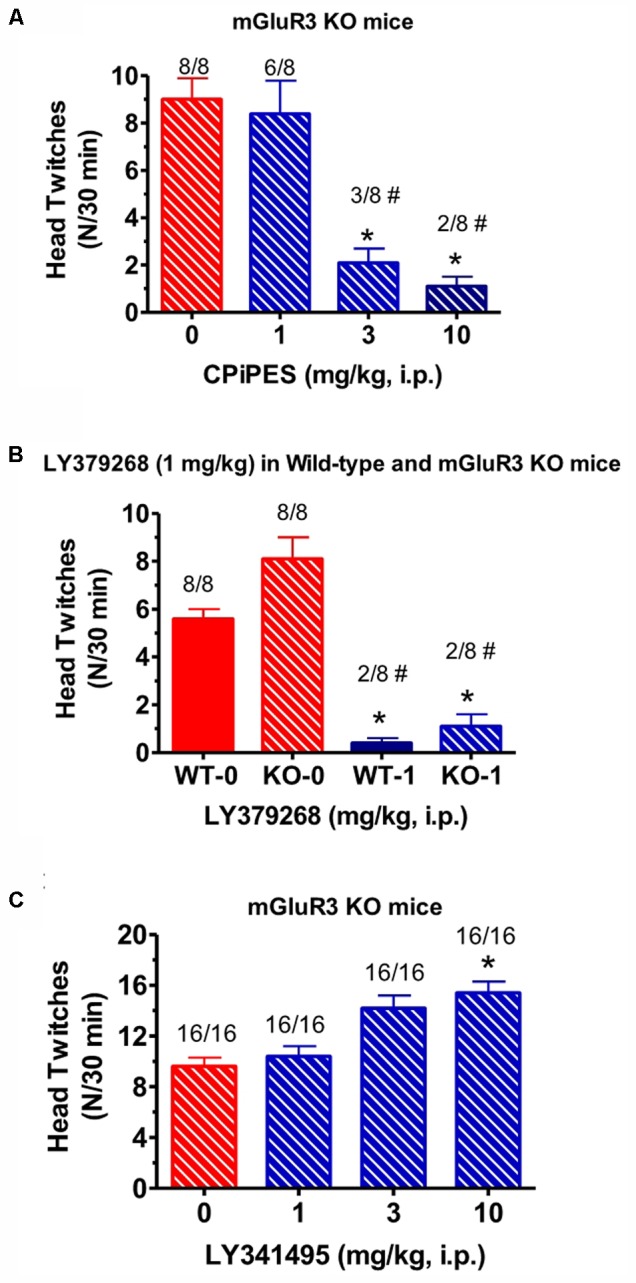
**(A)** Effect of CBiPES (1–10 mg/kg) on head twitches induced by DOI in mGlu_3_ receptor KO mice for 30 min following drug administration. CBiPES was administered 30 min prior to DOI. Each bar represents the mean (±SEM) of eight animals after i.p. dosing. Significantly different from 0 or the vehicle condition, ^∗^*p* < 0.05. Significantly different from 0 (vehicle) by the Fishers Exact Test, ^#^p < 0.05. **(B)** Effect of LY379268 (1 mg/kg) on head twitches induced by DOI in mGlu_3_ receptor KO mice for 30 min following drug administration. LY379268 was administered 30 min prior to DOI. Each bar represents the mean (±SEM) of eight animals after i.p. dosing. Significantly different from respective WT, ^∗^*p* < 0.05 or KO, ^∗∗∗^*p* < 0.05 treated only with DOI. Significantly different from respective WT and KO treated only with DOI by Fishers Exact Test, ^#^*p* < 0.05. **(C)** The effect of LY341495 (1–10 mg/kg) on head twitches induced by DOI in mGlu_3_ receptor KO mice for 30 min following drug administration. LY341495 was administered 30 min prior to DOI. Each bar represents the mean (±SEM) of 16 animals after i.p. dosing. Significantly different from DOI alone (“0” condition in the figure), ^∗^*p* < 0.05.

When the mGlu_2_ receptor PAM CBiPES (1–10 mg/kg) was injected in mGlu_3_ receptor KO mice, it produced a significant decrease in mean head twitches [*F*(3,28) = 5.2, *p* < 0.05] at doses of 3 and 10 mg/kg compared to vehicle/DOI-treated animals (**Figure 3A**). The number of animals emitting head twitches at those doses was also significantly reduced (3/8, 2/8, respectively). A CBiPES dose of 1 mg/kg was ineffective in suppressing the DOI-induced HTR. When the orthosteric mGlu_2/3_ receptor agonist LY379268 (1 mg/kg) was administered to mGlu_3_ receptor KO mice following DOI, the effect was the same as seen in the WT animals (**Figure 3B**). LY379268, at a dose of 1 mg/kg was fully effective [*F*(3,60) = 2.82, *p* < 0.05] at attenuated head twitches in both the mGlu_3_ KO and the WT mice. When the mGlu_2/3_ receptor antagonist LY341495 (1–10 mg/kg) was tested in mGlu_3_ receptor KO mice, there was a dose-dependent increase in mean head twitches tested (1–10 mg/kg; **Figure 3C**), an effect that reached statistical significance [*F*(3,60) = 2.82, *p* < 0.05]. *Post hoc* analysis revealed that the 10 mg/kg dose produced a significant increase in mean head twitches as compared to vehicle-treated animals (*p* = 0.046) and lower doses were not significant different from DOI alone.

### Drug Combinations in mGlu_3_ Receptor KO Mice

**FIGURE 4:**
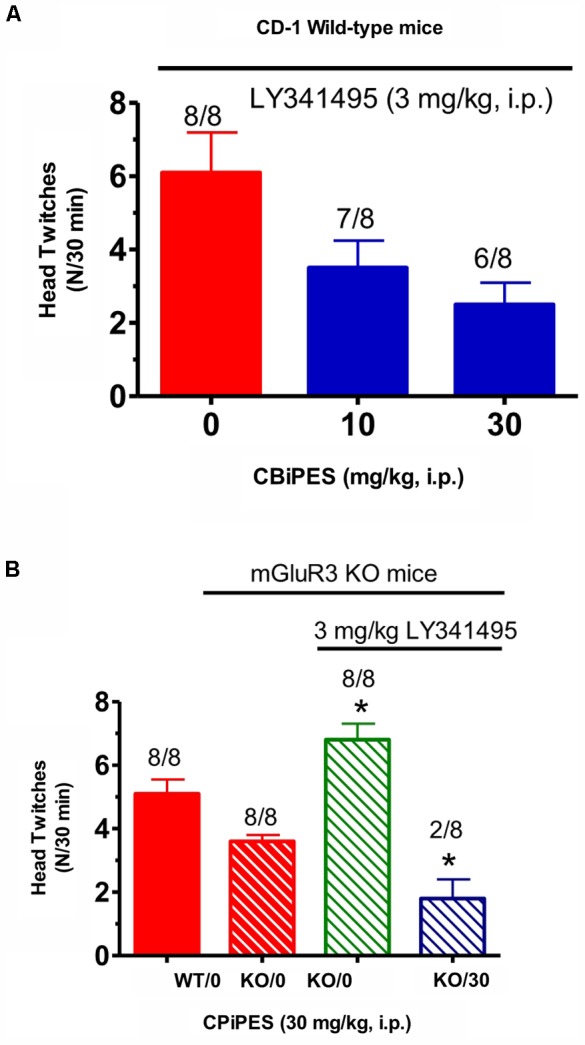
**(A)** The effect of vehicle or CBiPES (10–30 mg/kg) on head twitches induced by the administration of DOI (3 mg/kg) and LY341495 (3 mg/kg) in CD-1 WT mice for 30 min following drug administration. CBiPES was administered 5 min prior to LY341495, which was administered 30 min prior to DOI. Each bar represents the mean (±SEM) of eight animals after i.p. dosing. **(B)** The effect of vehicle or CBiPES (30 mg/kg) on head twitches induced by DOI (3 mg/kg), with or without the presence of LY341495 (3 mg/kg), in WT and mGlu_3_ receptor KO mice for 30 min following drug administration. Each bar represents the mean (±SEM) of eight animals after i.p. dosing. KO-LY341495 significantly different from KO without LY341495, ^∗^*p* < 0.05 or KO-DOI-LY341495-CBiPES significantly different from KO-DOI-LY341495, ^∗^*p* < 0.05.

Tests for the combination of the mGlu_2_ receptor potentiator, CBiPES and the mGlu_2/3_ receptor antagonist LY341495, can be seen in **Figure 4**. When DOI (3 mg/kg) and LY341495 (3 mg/kg) were dosed in WT mice, the addition of 30 mg/kg of CBiPES (**Figure 4A**) reduced the mean head twitches by 60% while 10 mg/kg of CBiPES reduced mean head twitches by almost 50%. However, this effect did not reach statistical significance [*F*(2,21) = 1.23, *p* = 0.31]. When the same dosing regimen was given to mGlu_3_ receptor KO mice (**Figure 4B**), LY341495 (3 mg/kg) significantly increased mean head twitches in the animals compared to mGlu_3_ receptor KO mice given DOI alone (*t* = 2.52, *p* < 0.05), and when CBiPES (30 mg/kg) was added to DOI + LY341495 (3 mg/kg), mean head twitches were significantly reduced by over 80% (*t* = 5.4, *p* < 0.05), and only two of eight mice emitted head twitches while eight of eight animals emitted head twitches when given vehicle + DOI + LY341495. So, while the addition of the mGlu_2/3_ receptor antagonist increased the number of head twitches produced by DOI in mGlu_3_ receptor KO mice, CBiPES, the mGlu_2_ receptor potentiator was able to attenuate the increase produced by the orthosteric antagonist LY341495.

## Discussion

The most striking finding from the present results was the dramatic rightward shift in the DOI-induced HTR in transgenic mice lacking mGlu_2_ receptors compared to either CD-1 mice, WT mice or transgenic mice lacking mGlu_3_ receptors. A monotonic increasing DOI-induced HTR was observed in CD-1 mice over a 1–10 mg/kg dose range. In contrast to this, over a 10-fold rightward shift in the dose-response relationship was observed in mGlu_2_ KO mice. No head twitches were observed in the mGlu_2_ KO mice for a 3 mg/kg DOI dose while only three of eight mice exhibited head twitches at the 30 mg/kg DOI dose. This finding is consistent with the relative loss of DOI-induced head twitches using a different line of mGlu_2_ receptor KO mice (Moreno et al., 2011), but extends this finding by examining a wider dose range. This marked rightward shift in the DOI-induced HTR in mGlu_2_ KO mice has also been confirmed by a pharmacological experiment where subchronic daily treatment (21 days) with the mGlu_2/3_ receptor antagonist LY341495 also down-regulated 5-HT_2A_ receptor density and the frequency of DOI-induced head twitches in WT mice (Moreno et al., 2013).

**FIGURE 5:**
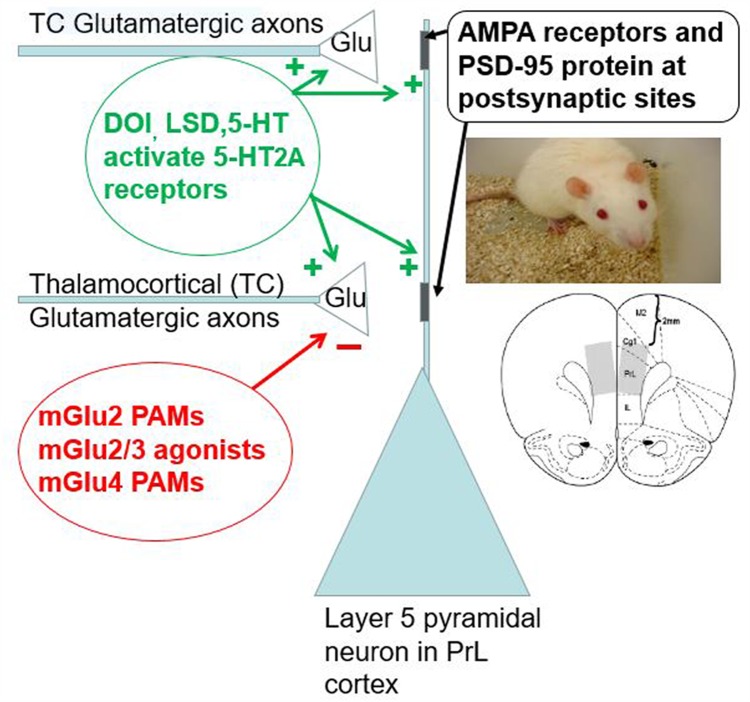
The range of serotonergic and glutamatergic modulation in the layer V pyramidal neuron apical dendritic field from the prefrontal cortex/neocortex is shown. 5-HT_2A_ receptors are present at postsynaptic sites to glutamatergic axon terminals and may also be present at presynaptic thalamocortical axon terminals (arising from the midline and thalamic nuclei) relative to the layer V pyramidal neuron apical dendrites. 5-HT_2A_ receptor activation appears to induce glutamate release from these midline and intralaminar thalamic nuclei axons. Due to the presence of both mGlu_2_ receptors acting as a critical autoreceptor on these thalamocortical terminals, mGlu_2/3_ receptor agonists and mGlu_2_ receptor PAMs suppress glutamate release from these terminals in a homeostatic fashion. MGlu_4_ receptors also may be present on these glutamatergic terminals. Glutamate released by these thalamocortical axons activates AMPA receptors on the layer V pyramidal neuron apical dendrite. The PSD-95 protein is a scaffolding protein that may keep AMPA and postsynaptic 5-HT_2A_ receptors in close physical proximity. The lower insert shows a coronal slice of the mouse brain including the prelimbic region of the medial prefrontal cortex. This region appears to support DOI-induced head twitches in rodents. Thus, this microcircuitry appears very salient for rodent behavior, at least with respect to DOI-induced head twitches.

This finding of the near loss of DOI-induced HTR in mGlu_2_ KO mice was previously suggested to be related to heteromeric 5-HT_2A_/mGlu_2_ receptors in the prefrontal cortex (Moreno et al., 2011, 2012). However, other interpretations are also plausible. The most likely alternate hypothesis is a down-regulation in the sensitivity of 5-HT_2A_ receptors to agonist stimulation mediating the desensitization of serotonergic hallucinogen-induced head twitches. A single large dose or subchronic smaller doses of phenethylamine hallucinogens or LSD is known to decrease 5-HT_2A_ receptor binding in rodents (Buckholtz et al., 1985, 1988; Leysen et al., 1989; Shi et al., 2008; Buchborn et al., 2015). Accordingly, either multiple injections within a single day or daily dosing over a week result in a tachyphylaxis for hallucinogen-induced head twitches (Leysen et al., 1989; Darmani et al., 1992; Darmani and Gerdes, 1995; Buchborn et al., 2015). Serotonergic hallucinogens have been shown to increase extracellular cortical glutamate in rodents (Scruggs et al., 2003; Muschamp et al., 2004). These three types of serotonergic hallucinogen induced effects are consistent with the finding that a single dose of the mGlu_2/3_ receptor agonist LY354740 was able to reverse to decrease in prefrontal cortical DOI binding from a subacute regimen of 3 daily DOI doses (Marek et al., 2006). Furthermore, just 3–4 days following an adenoviral incorporation of functional mGlu_2_ receptors in the prefrontal cortex of mGlu_2_ receptor KO mice restored a normal appearing DOI-induced HTR (Moreno et al., 2012). Conversely, lesions of the glutamatergic input to the prefrontal cortex and neocortex from the midline and intralaminar thalamic nuclei, along with most other thalamic inputs, resulted in an approximately 20% increase in DOI binding several weeks following the chemical lesion of the thalamus (Marek et al., 2001). This implies that at least some effects of cortical 5-HT_2A_ receptor function/regulation occurs within a context of glutamatergic input from the intralaminar and midline thalamic nuclei (Scruggs et al., 2000; Lambe and Aghajanian, 2001; Marek et al., 2001) as modified by mGlu_2_ autoreceptors. The critical role of mGlu_2_ receptors as an autoreceptor on thalamocortical glutamatergic afferents to layer V pyramidal cell apical dendrites appears to provide a biological substrate where homeostasis exists for optimal stimulation of serotonergic and glutamatergic drive at layer V pyramidal cell apical dendrites. The paradoxical effects between acute and chronic perturbation may be a consequence of maintaining a homeostatic state of circuit activity (**Figure 5**).

A second major finding is that both the suppressant action of a mGlu_2/3_ receptor agonist and a mGlu_2_ receptor PAM on the DOI-induced HTR was retained in mGlu_3_ receptor KO mice. Unfortunately, the large rightward shift in the DOI-induced HTR in mGlu_2_ KO mice effectively precluded testing the effects of mGlu_2/3_ receptor agonists or mGlu_2_ receptor PAMs in the mGlu_2_ receptor KO mice. The down-regulation of DOI-induced head twitches in mGlu_2_ receptor KO mice coupled with the finding that the suppressant effects of mGlu_2/3_ receptor agonists or mGlu_2_ receptor PAMs on the DOI-induced HTR are present in mGlu_3_ receptor KO mice is consistent with the hypothesis that activation of mGlu_2_ receptors may be a necessary and sufficient pharmacological action when mGlu_2/3_ receptor agonists suppress the DOI-induced HTR. However, a subtle role of mGlu_3_ receptors in modulating the DOI-induced HTR would require testing of selective mGlu_3_ receptor agonists or PAMs. Nevertheless, the mGlu_2_ receptor KO finding replicates observations with an independent mouse transgenic strain lacking mGlu_2_ receptors (Moreno et al., 2011).

Previously, acute treatment with a mGlu_2/3_ receptor antagonist was found to increase the mean DOI-induced HTR in rats (Gewirtz and Marek, 2000). This effect was present in mGlu_3_ receptor KO mice, suggesting this increased behavioral DOI response is due to blockade of presumed mGlu_2_ autoreceptors on thalamic inputs to the cortex. The failure to similarly observe a significant increase in DOI-induced head twitches following administration of a mGlu_2/3_ receptor antagonist in WT mice may have been related to examining a limited portion of the dose-response relationship (only 1–3 mg/kg of LY341495 for the WT experiment vs. 1–10 mg/kg for the mGlu_3_ receptor KO experiment) or a smaller sample size (16 vs. 8).

The robust rightward shift in the DOI dose-response relationship for the DOI-induced HTR in mice lacking mGlu_2_ receptors implies that decreasing feedback regulation of glutamate release plays a major role in the regulation of at least some cortical 5-HT_2A_ receptor responses. Another independent transgenic line also shows a robust decrease in the DOI HTR (Moreno et al., 2011). Mice lacking the serotonin transporter (SERT) respond to DOI with minimal if any head twitches (Qu et al., 2005; Fox et al., 2010), in keeping with the critical importance of SERT for inactivating the synaptic 5-HT signal by reuptake of 5-HT back into the presynaptic terminal. Without SERT, the 5-HT_2A_ receptor is down-regulated by the supra-physiological neuropil 5-HT concentrations.

Conversely, a robust up-regulation or leftward shift in the DOI-induced HTR has also previously been observed in a mutant mouse strain lacking a number of presynaptic 5-HT components (tryptophan hydroxylase2, the serotonin transporter, the vesicular monoamine transporter 2, and aromatic amino acid decarboxylase) (Yadav et al., 2011). Brain 5-HT concentrations for these *pet1*^-/-^ mutant mice is only 15% of WT mice (Hendricks et al., 2003). These *pet1*^-/-^ mice exhibit an overactivity of 5-HT_2A_ receptor behavioral function in the prefrontal cortex that is probably due to a decrease in tonic inhibitory 5-HT_1A_ receptor activity (Yadav et al., 2011).

In addition to the presynaptic side of either serotonergic or glutamatergic terminals appearing to cause a profound regulation of the DOI-induced HTR, mutant mice lacking the postsynaptic density protein of 95 kDa (PSD-95), a scaffolding protein associated with glutamatergic synapses, show an attenuated DOI-induced HTR (Abbas et al., 2009). This may be related to a PSD-95 binding motif in the 5-HT_2A_ receptor protein that is postulated to play a role in dendritic targeting of 5-HT_2A_ receptors (Xia et al., 2003a,b). These relationships between presynaptic modulation of both glutamate and serotonin release as well as the postsynaptic side machinery including AMPA receptors (Zhang and Marek, 2008) and 5-HT_2A_ receptors highlights a critical role played by cortical 5-HT_2A_ receptor activity in regulating the output of the prefrontal cortex/neocortex (**Figure 5**).

A limitation of the present findings may be an underestimation of the total number of head twitches observed during the observation period. For example, the frequency of DOI-induced head twitches may be about two–threefold lower than other examples recent examples of the more frequently used observation of a single mouse at a time (Griebel et al., 2016). This study may also be on the low side compared to other studies cited in the introduction. A second limitation of the study is the inherent variability in the DOI-induced HTR. A two–threefold variation in the mean number of DOI-induced HTR between different experiments was observed in the present studies as well. Other experiments from our laboratory in Sprague-Dawley rats, with mean head twitch frequencies induced by DOI (3 mg/kg, i.p.) in the range of reports using single observations, have found that two mGlu_2_ receptor PAMs (CBiPES and THIIC) significantly suppressed the frequency of DOI-induced head twitches (**Supplementary Figure S1**). Similarly, the only other proprietary mGlu_2_ receptor PAM that was tested in both WT and mGlu_3_ receptor KO mice demonstrated reliable effects of both DOI and the suppressant action of this mGlu_2_ receptor PAM in both the WT and mGlu_3_ receptor KO mice (**Supplementary Figure S2**). In addition, replicable effects of DOI alone in a series of 12 experiments with proprietary mGlu_2_ receptor PAMs (see description of the head twitch test in the section “Materials and Methods”) does not suggest that there is a major role for tachyphylaxis of the DOI-induced HTR when employing the currently described methodology in WT mice. Most importantly, the pattern of results obtained with mGlu_2/3_ receptor agonists, mGlu_2_ receptor PAMs, and mGlu_2/3_ receptor antagonists in the present experiments is in good agreement with past results for these compounds in rodents and mice as discussed in the Section “Introduction.”

Clinical implications for these relationships between 5-HT_2A_ receptors and mGlu_2_ receptors are multifold including the notion that these relationships may paradoxically limit clinical efficacy for 5-HT_2A_ or mGlu_2_ receptor ligands. First, the antipsychotic-like properties of 5-HT_2A_ receptor inverse agonists or antagonists were clearly demonstrated for patients with Parkinson’s disease psychosis (Meltzer et al., 2010; Cummings et al., 2014). This replicated earlier findings that 5-HT_2A_ receptor antagonists exerted modest antipsychotic properties in patients with schizophrenia (Marder, 1999; Meltzer et al., 2004), in keeping with psychotomimetic effects of LSD, mescaline and psilocybin. Secondly, a role for mGlu_2_ receptor agonists or PAMs for the treating psychosis is another implication derived in part from a range of interactions between 5-HT_2A_ and mGlu_2_ receptors. However, antipsychotic efficacy of mGlu_2/3_ agonists or mGlu_2_ receptor PAMs may be restricted to those subjects with schizophrenia not previously exposed to atypical antipsychotics with nearly complete 5-HT_2A_ receptor blockade or in those subjects early in their personal trajectory with schizophrenia (Patil et al., 2007; Kinon et al., 2015). Given the pimavanserin clinical profile in Parkinson’s disease psychosis, mGlu_2_ receptor agonists or PAMs might be useful in patients with neurodegenerative disease suffering from psychotic-like symptoms. Ironically, the earlier hope that the extensive relationships between 5-HT_2A_ receptors and mGlu_2_ receptors would provide a path toward therapeutic drugs with broad antipsychotic clinical activity may instead mean that antipsychotic efficacy with mGlu_2_ receptor agonists/PAMs might be restricted to a smaller subpopulation of patients.

## Author Contributions

MJB and GM designed the experiments and wrote the manuscript. TB, WH, and JM synthesized the mGlu receptor drugs. MB developed and maintained the transgenic mice. SC performed the experiments. MJB, JM, and GM analyzed and discussed the data.

## Conflict of Interest Statement

All work was funded by Eli Lilly and Co. and was performed at Lilly Neuroscience Discovery laboratories. The authors declare that the research was conducted in the absence of any commercial or financial relationships that could be construed as a potential conflict of interest.

## Supplementary Material

**FIGURE S1 |** Two mGlu_2_ receptor positive allosteric modulator (PAMs) (CBiPES and THIIC) suppress DOI-induced head twitches in Sprague-Dawley rats. Both DOI (3 mg/kg) and the mGlu_2_ receptor PAMs were administered i.p. CBiPES and THIIC were injected 30 min prior to DOI and then the eight animals/dose cohort were observed immediately afterward for 30 min. The rats were approximately 5–6 weeks old (100–150 g). The individual chamber size was 25% larger in both length and width than the mouse chambers. Eight rats were observed at a single time. The top graph shows the effects of CBiPES while the lower graph provides the results of the THIIC experiment. Each bar represents the mean (±SEM) of eight rats. ^∗^Significantly different from the DOI alone (0) group, *p* < 0.05. Red bars display the effects of DOI alone while the blue bars show the effects of different doses of the mGlu_2_ receptor PAMs.

**FIGURE S2 |** The suppressant effect of a mGlu_2_ receptor PAM, compound A, on DOI-induced head twitches in wild-type CD1 mice (top graph) and also in both wild-type and mGlu_3_ receptor KO mice bottom graph). Compound A was administered 30 min prior to DOI. Each bar represents the mean (±SEM) of eight animals after i.p. dosing. Significantly different from 0 or the vehicle condition, ^∗^*p* < 0.05. Significantly different from 0 (vehicle) by the Fishers Exact Test, ^#^*p* < 0.05. The solid red bars show the effects of DOI alone in the wild-type mice while the solid blue bars display the suppressant effect of compound A on DOI-induced head twitches. The red hatched bars show the effects of DOI alone in the mGlu_3_ receptor KO mice while the blue hatched bars display the suppressant effect of compound A on DOI-induced head twitches. Significantly different from respective WT, ^∗^*p* < 0.05 or KO, ^∗^*p* < 0.05 treated only with DOI. Significantly different from respective WT and KO treated only with DOI by Fishers Exact Test, ^#^*p* < 0.05.
