# Supplementary figures and images for: Metabotropic Glutamate2 Receptors Play a Key Role in Modulating Head Twitches Induced by a Serotonergic Hallucinogen in Mice
Source: Front Pharmacol. 2018 Mar 15;9:208. doi: 10.3389/fphar.2018.00208 (PMC5862811; doi:10.3389/fphar.2018.00208)

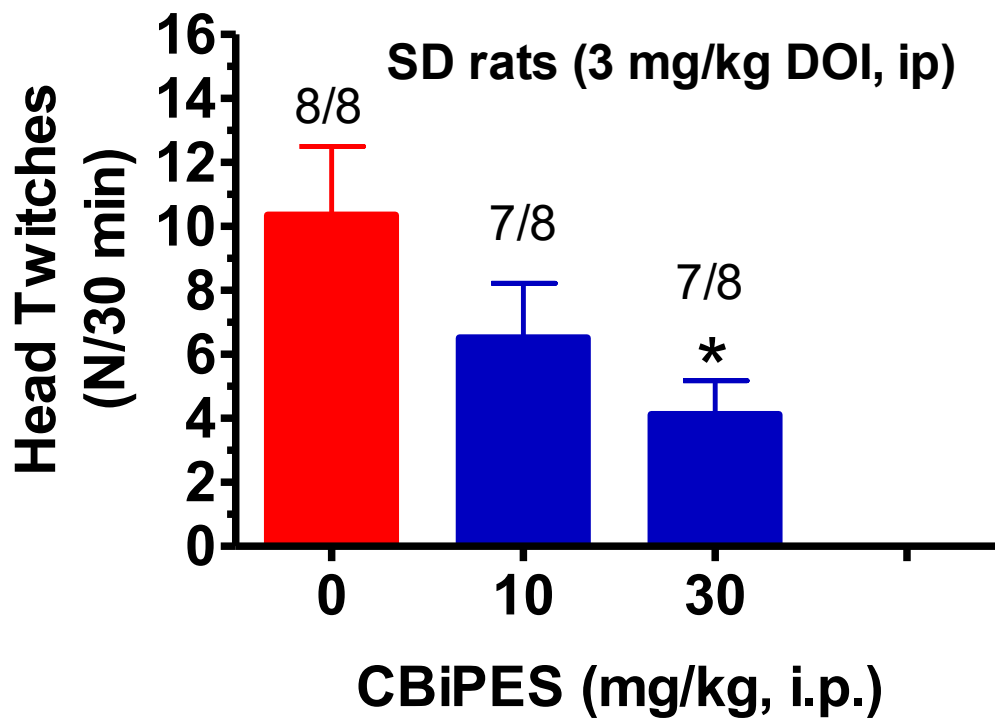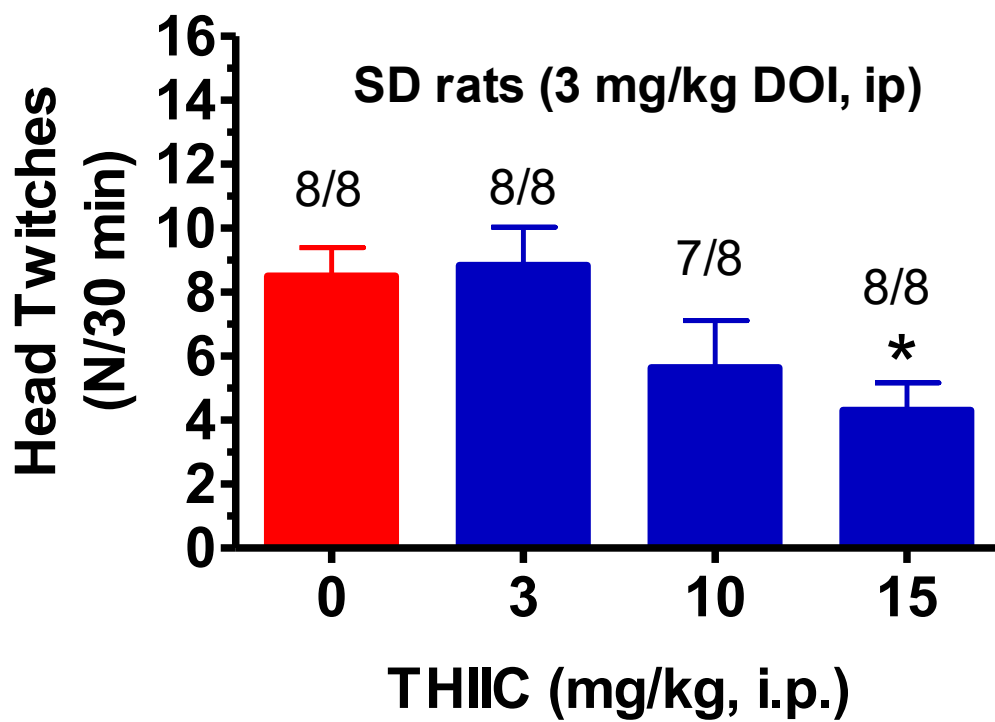

Supplement: Supplementary file 1 [file Data_Sheet_1.PDF]

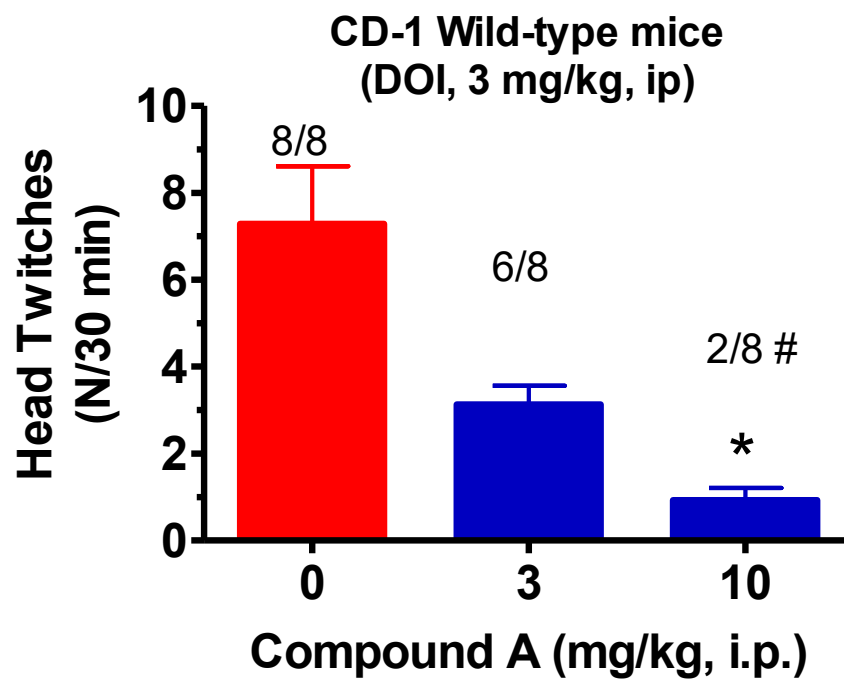

**Compound A (1 mg/kg) in Wild-type and mGluR3 KO mice**

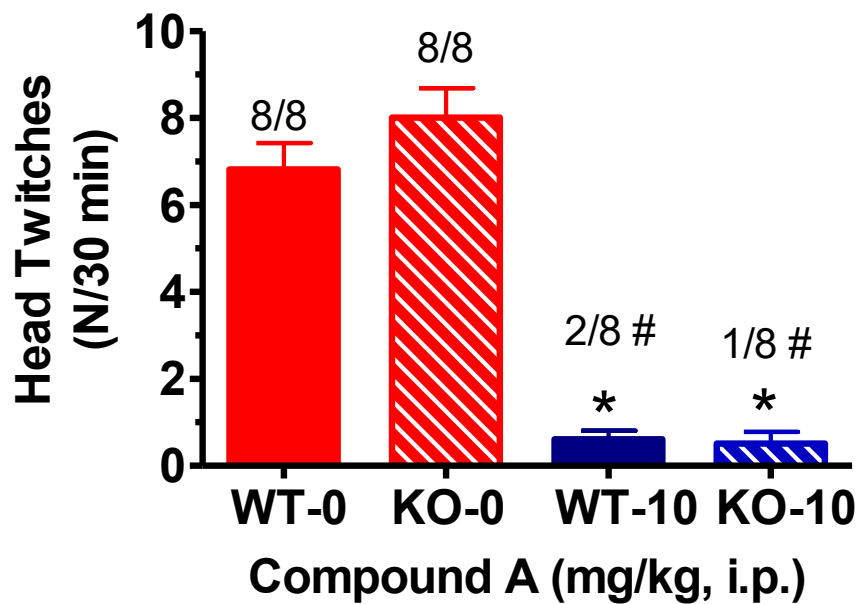

Supplement: Supplementary file 2 [file Data_Sheet_2.PDF]
